# Supplementary material for: A Recombinant Multivalent Vaccine (rCpa1) Induces Protection for C57BL/6 and HLA Transgenic Mice against Pulmonary Infection with Both Species of Coccidioides
Source: Vaccines (Basel). 2024 Jan 9;12(1):67. doi: 10.3390/vaccines12010067 (PMC10819930; doi:10.3390/vaccines12010067)
Supplement: Supplementary file 1 [file vaccines-12-00067-s001.zip › vaccines-2749047-supplementary.pdf]

Supplemental Figure S1. Alignment of rCpa1 and rCpa2

rCpa1 accession no. KY883768, 586 AA

rCpa2 accession no. WAB54687.1, 586 AA

CLUSTAL O(1.2.4) multiple sequence alignment

```
rCpa1      MGSSHHHHHHSSGLVPRGSHMGPGPGMQFSHALIALVAAGLASAQLPDIPPCALNCFVEA   60
rCpa2      MGSSHHHHHHSSGLVPRGSHMGPGPGMQFSHALIALVAAGLASAQLPDIPPCALNCFVEA   60
           ************************************************************

rCpa1      LGNDGCTRLTDFKCHCSKPELPGQITPCVEEACPLDARISVSNIVVDQCSKAGVPIDIPP   120
rCpa2      LGNDGCTRLTDFKCHCSKPELPGQITPCVEEACPLDARISVSNIVVDQCSKAGVPIEIPP   120
           ********************************************************:***

rCpa1      VDTTAAPEPSETGPGPGMKFSLLSAIAAAVFVPFTSATPLASTADLSYDTHYDDPSLPLS   180
rCpa2      VDTTAAPEPSETGPGPGMKFSLLSAIAAAVFVPFTSATPLASTTDLSYDTHYDDPSLALS   180
           *******************************************:************* **

rCpa1      GVTCSDGDNGMITKGYNTAGEIPNYPHVGGAFTVETWNSPNCGKCYKVTYNAKTIFLTAI   240
rCpa2      GVTCSDGDNGMITKGYNTAGEIPNYPHVGGAFTVETWNSPNCGKCYKVTYNAKTIFLTAI   240
           ************************************************************

rCpa1      DHSNSGFNIAKKSMDVLTNGRAEELGRIKVTYEEVASSLCGLKGPGPGMASLKAGDSFPS   300
rCpa2      DHSNSGFNIAKKSMDVLTNGRAEELGRIKVTYEEVASSLCGLKGPGPGMASLKAGDSFPS   300
           ************************************************************

rCpa1      DVVFSYIPWTPDNKDIKACGMPQNYEASKLWADKKVVLFSLPGAFTPTCSASHLPGYIQK   360
rCpa2      DVVFSYIPWTPDNKDIKACGMPQNYEASKLWADKKVVLFSLPGAFTPTCSASHLPGYIQK   360
           ************************************************************

rCpa1      LPQLKEKGVDVVAVLAFNDAWVMSAWGKANGVTGDDILFLSDPEAKFSKSIGWNAGERTG   420
rCpa2      LPQLKEKGVDVVAVLAFNDAWVMSAWGKANGVTGDDILFLSDPEAKFSKSIGWNAGERTG   420
           ************************************************************

rCpa1      RYAMIIDHGQVTYAEIEPGREVTVSGADAVISKLGPGPGMRNSILLAATVLLGCTSAKVH   480
rCpa2      RYAIIIDHGKVTYAEIEPGREVTVSGADAVFSKLGPGPGMRNSILLAATVLLGCTSAKVH   480
           ***:*****:********************:*****************************

rCpa1      GPGPGHVRALGQKYFGSLPSSQQQTVGPGPGPAKVDVLLAQSLKLADVLKFGPGPGNGLA   540
rCpa2      GPGPGHVRALGQKYFGSLPSSQQQTVGPGPGPAKVDVLLAQSLKLADVLKFGPGPGNGLA   540
           ************************************************************

rCpa1      TTGTLVLEWTRLSDITGPGPGTPLVVYIPNYPYTTWSNISTGPGPG   586
rCpa2      TTGTLVLEWTRLSDITGPGPGTPLVVYIPNYPYTTFSNISTGPGPG   586
           ***********************************:**********
```

Supplementary Table S1. Primer sequences and predicted sizes of PCR amplicons of the *Coccidioides* antigens

Antigen GenBank No.	Primer Sequences*	PCR amplicon size (bp)	Reference
Ag2/Pra (XM_003069107)	Forward: 5′-AATCGTTCTCGTCCGTTAGA-3′<br>Reverse: 5′-CTCCTCGGTTGGCTCAG-3′	585	Jiang et al, 1999<br>Peng et al, 1999<br>Kirkland et al, 1998<br>Herr et al, 2007
Cs-Ag (XM_003065932)	Forward: 5′-GGGAAAGATGAAGTTCTCACTCCT-3′<br>Reverse: 5′-GCACATCGCCCAATACGCCTTTA-3′	443	Pan et al, 1995<br>Shubitz et al, 2006
Pmp1 (XM_003069228)	Forward: 5′-GTCTGCCAACGCTCGATTCAC-3′<br>Reverse: 5′-GGCCGTTTCCCTTGATTCTCC-3′	501	Orsborn et al, 2006
Amn1 (XM_003068811)	Forward: 5′-TGAAACCACCATTCGCTACCT-3′<br>Reverse: 5′-AGGCCCGTCTCAATGTCAAT-3′	331	Tarcha et al, 2006<br>Hurtgen et al, 2012<br>Hurtgen et al, 2017
Plb (XM_003065040)	Forward: 5′-CTTTGCCATCGACTCTTCTG-3′<br>Reverse: 5′-CAAACGACCGACTCAAGATAG-3′	677	
Pep1 (XM_001244245.2)	Forward: 5′-CACACGCTCTTTCAAGACC-3′<br>Reverse: 5′-CAGAGATTTGAGCTTCCTGTG-3′	537	

*Gene-specific primers only flank DNA fragments of each antigenic peptides that were incorporated in the chimeric rCpa1 vaccine antigen (GenBank accession AVH85517.1).
